# Profil clinique et survie des patients avec néphrite lupique en néphrologie au Cameroun: étude monocentrique

**DOI:** 10.11604/pamj.2022.41.205.28844

**Published:** 2022-03-14

**Authors:** Hermine Danielle Fouda Menye Ebana, Maimouna Mahamat, Fernando Kemta Lekpa, Caroline Kenmegne Jemmy, Gloria Ashuntantang, Marie-Patrice Halle

**Affiliations:** 1 Hôpital Général de Douala, Douala, Cameroun,; 2 Faculté de Médecine et des Sciences Biomédicales de Yaoundé, Yaoundé, Cameroun,; 3 Hôpital Général de Yaoundé, Yaoundé, Cameroun,; 4 Faculté de Médecine et des Sciences Pharmaceutiques de Dschang, Dschang, Cameroun,; 5 Faculté de Médecine et des Sciences de la Santé de Bamenda, Bamenda, Cameroun,; 6 Faculté de Médecine et des Sciences Pharmaceutiques de Douala, Douala, Cameroun

**Keywords:** Néphrite lupique, syndrome néphrotique, rémission, survie, Lupus nephritis, nephrotic syndrome, remission, survival

## Abstract

**Introduction:**

le pronostic de la néphrite lupique en Afrique Subsaharienne est mal connu. Notre objectif était de décrire le profil clinique et la survie des patients avec une néphrite lupique suivis en néphrologie.

**Méthodes:**

nous avons mené une étude de cohorte rétrospective mono centrique sur 5 ans. Les patients avec une néphrite lupique nouvellement diagnostiquée et un suivi de plus de 3 mois ont été inclus. La néphrite lupique était définie par la présence de signes d´atteinte glomérulaire, confirmée ou non à l´histologie. Nous nous sommes intéressés aux données cliniques, à l´évolution sous traitement et à la mortalité.

**Résultats:**

au total, 20 patients ont été inclus dont 17 femmes (85%). La médiane [IIQ] d´âge était de 27[18-37] ans. Le lupus érythémateux systémique était diagnostiqué concomitamment à la néphrite lupique chez 90% (n=18) des patients. Douze (60%) patients avaient un syndrome néphrotique. Les classes prolifératives actives étaient les plus fréquentes (n=5, 72%). Quinze patients (75%) ont bénéficié d´une induction et la rémission était obtenue chez 6 (30%). A 12 et 24 mois, les survies rénales et globales étaient respectivement de 68,6% et 49%; de 57,8% et 31%. L´absence de rémission était associée au mauvais pronostic.

**Conclusion:**

la néphropathie lupique est un mode de révélation fréquent du lupus dans notre contexte. Son pronostic est sombre et plus de la moitié des patients décèdent ou sont en insuffisance rénale terminale dans les 24 mois.

## Abstract (en)

**Introduction:**

prognosis of lupus nephritis in sub-Saharan Africa is poorly known. The purpose of this study is to describe the clinical profile and survival of patients with lupus nephritis treated in the Department of Nephrology.

**Methods:**

we conducted a single-centre retrospective cohort study over a period of 5 years. Patients with newly diagnosed lupus nephritis and followed-up for more than 3 months were included in the study. Lupus nephritis was defined as the presence of signs of glomerular damage, whether histologically confirmed or not. We investigated clinical data, treatment effects, and mortality rates.

**Results:**

a total of 20 patients were enrolled in the study, including 17 women (85%). The average age [IQ] of patients was 27 [18- 37] years. Systemic lupus erythematosus was diagnosed concomitantly with lupus nephritis in 90% (n=18) of patients. Twelve (60%) patients had nephrotic syndrome. Active proliferative classes were mainly reported (n=5, 72%). Fifteen patients (75%) received induction therapy and remission was obtained in 6 (30%) patients. At 12 and 24 months, renal and global survival was 68.6% and 49%, and 57.8% and 31%, respectively. The absence of remission was associated with poor prognosis.

**Conclusion:**

lupus nephropathy is a common way of revealing lupus in our context. Prognosis is poor, and more than half of patients die or develop end-stage renal disease within 24 months.

## Introduction

La néphrite lupique (NL) est une complication grave et fréquente du lupus érythémateux systémique (LES). Son incidence et sa prévalence varient selon la population étudiée avec des incidences cumulées plus importantes chez les patients d´origine Africaine et Asiatique, les sujets de sexe masculin, les adolescents et les patients de faible niveau socio-économique [1-3]. L´insuffisance rénale chronique terminale (IRCT) est la complication la plus redoutée de la NL. En effet, 10% des patients avec une NL sont susceptibles de développer une IRCT. Le risque de décès des patients avec une NL est 6 fois plus élevé que dans la population générale avec les infections et les décès cardiovasculaires comme principales causes de mortalité [4]. Par ailleurs, le coût annuel de la NL aux Etats-Unis est estimé entre 43.000 et 107.000 dollars [4,5]. Dans les pays développés, les avancées thérapeutiques dans la prise en charge de la NL, notamment des formes prolifératives, ont permis l´amélioration du pronostic de la NL avec une diminution du risque IRCT à 5 ans de 16% (95% IC 14-17%) de 1970-1979 à 11% (95% IC 10-12%) depuis 1995 et des survies globales actuelles à 5 ans de 98,2% [6]. La NL est également décrite dans les régions à ressources limitées, notamment en Afrique Subsaharienne. En Afrique du Sud, la NL est retrouvée chez 43,8% des patients ayant un LES et constitue le principal facteur de risque de décès [7]. Au Sénégal, la fréquence de la NL est estimée entre 69 et 72% avec un taux de mortalité entre 7,8 et 9,3% [8,9]. Au Cameroun, peu d´études se sont intéressées à la NL. Une série de 39 cas de LES retrouvait que 18% des patients avaient d´emblée une atteinte rénale au diagnostic du LES et l´IRCT était la principale complication et la principale cause de décès [10]. Toutefois, les aspects cliniques et le pronostic de la NL dans notre contexte restent mal connus. L´objectif de cette étude était de décrire les aspects cliniques et les survies rénales et globales des patients avec une NL à l´Hôpital Général de Douala (Cameroun).

## Méthodes

### Type et lieu de l´étude

Nous avons mené une étude de cohorte rétrospective sur 5 ans (1^er^ janvier 2015 au 31 décembre 2020) dans le service de néphrologie de l´Hôpital Général de Douala (HGD). Il s´agit du principal service de Néphrologie de la ville de Douala (ville la plus peuplée du Cameroun) avec plus de 650 nouveaux patients par an. Ce service abrite également le seul centre d´hémodialyse publique de la région du Littoral. Le service travaille en collaboration avec le service de Médecine Interne qui comporte entre autres des unités de Rhumatologie, de Dermatologie et de Neurologie.

### Participants

Les dossiers des patients avec un LES vus en consultation externe de néphrologie ont été colligés. Nous avons inclus les patients qui présentaient une atteinte rénale relevant un LES ou au décours d´un LES récemment diagnostiqué (< 6 mois). Les patients qui présentaient des signes en faveur d´une atteinte non glomérulaire et ceux qui étaient perdus de vue avec un suivi de moins de 3 mois ont été exclus. Nous nous sommes intéressés aux données démographiques (âge, sexe, niveau socio-économique), aux données cliniques et paracliniques initiales (comorbidités, pression artérielle, syndrome néphrologique, signes extra-rénaux, délai de découverte de LES, classe histologique) et aux données thérapeutiques (hydroxychloroquine, corticoïdes, immunosuppresseurs, traitement néphroprotecteur), à l´évolution de l´atteinte rénale (rémission/résistance, survenue IRCT) et la survenue du décès. Le revenu mensuel moyen et le niveau d´éducation ont été utilisés pour déterminer le niveau socio-économique des patients. Les patients vivants et sans IRCT à la fin du suivi étaient considérés comme ayant un bon pronostic. Les patients décédés ou en IRCT à la fin du suivi étaient considérés comme ayant un mauvais pronostic. Les définitions suivantes ont été utilisées: Lupus érythémateux systémique: le LES était diagnostiqué sur la base des critères de l´EULAR/ACR 2019 [11]. Néphrite lupique: la NL était définie par la présence de signe d´atteinte glomérulaire confirmée ou non à l´histologie et classée selon les critères 2018 de l´ISN/RPS. La maladie rénale chronique (MRC) était définie et classifiée selon les critères de la KDIGO 2012. L´IRCT correspondait au stade 5 de la MRC. La rémission était définie selon les critères de la Kidney Disease Improving Global Outcomes (KDIGO) et évaluée après 6-12 mois de traitement. Les patients qui présentaient une protéinurie < 0,5g/g et une normalisation de leur créatininémie (± 10-15% de la créatininémie de base) avaient une rémission complète; ceux qui présentaient une réduction de leur protéinurie de plus de 50% avec une protéinurie < 3g/g et une créatininémie stable (± 25% de la créatininémie de base) avaient une rémission partielle et ceux qui ne remplissaient pas les critères de rémission complète ou partielle après 12 mois étaient considérés comme ayant une résistance.

### Considérations éthiques

Nous avons obtenu l´autorisation du comité d´éthique de l´HGD. Par ailleurs, l´étude a été menée dans le respect de principes fondamentaux en recherche humaine, notamment le respect de la personne humaine et les principes de bénéfice et de justice.

### Analyses statistiques

Les variables qualitatives ont été exprimées sous formes de proportion et de pourcentage. Les données quantitatives ont été présentées sous formes de moyenne ± écart type ou de médiane [Intervalle Interquartile (IIQ) 25e-75e] selon la distribution. Les patients ont été comparés selon leur pronostic. Les données qualitatives ont été comparées à l´aide du test exact de Fisher et les données quantitatives à l´aide du test Student ou de son équivalent non paramétrique. Les courbes de Kaplan-Meier ont été utilisées pour évaluer les survies rénales et globales et le test de Log-Rank pour les comparer. Le seuil de significativité était p < 0,05. Les données ont été analysées avec le logiciel IBM SPSS Statistics version 23.

## Résultats

Vingt-cinq dossiers de patients avec une atteinte rénale au cours d´un LES ont été identifiés sur 3800 nouvelles consultations. Cinq patients ont été exclus: 4 patients avaient une atteinte non glomérulaire (3 néphropathies tubulo-interstitielles et une micro angiopathie thrombotique) et 1 patient avait un suivi < 1 mois).

### Caractéristiques socio-démographiques

Des 20 patients inclus, 17 (85%) étaient des femmes. La médiane [IIQ] d´âge était de 27 [18-37] ans avec des extrêmes de 14 et 63 ans. Sept patients (33%) avaient un niveau socio-économique faible et la prise en charge des patients était personnelle ou familiale dans la majorité des cas (n=14, 70%) (Tableau 1).

**Tableau 1:** caractéristiques sociodémographiques des patients avec néphrite lupique colligés dans le service de néphrologie de l´Hôpital Général de Douala (Cameroun), de janvier 2015 à décembre 2020 (N=24)

Variables	Total (%) N=25	Bon pronostic (%) n=7	Mauvais pronostic (%) n=13	p
Age [IIQ] (année)	27 [18-38]	25 [20-25]	26 [18-38]	0,999*
**Sexe**				
Homme	3 (15)	-	3 (23)	-
Femme	17 (8)	7 (100)	10 (77)	
**Résidence**				
Urbaine	12 (60)	6 (86)	6 (46)	-
Semi-urbaine	5 (25)	-	5 (39)	
Rurale	3 (15)	1 (14)	2 (15)	
**Niveau socio-économique**				
Bas	7 (33)	2 (28,5)	5 (39)	0,459**
Moyen	10 (48)	3 (43)	7 (54)	
Élevé	4 (19)	2 (28,5)	1 (7)	
**Prise en charge**				
Personnel/famille	14 (70)	5 (71,5)	9 (69)	0,999**
Assurance	6 (30)	2(28,5)	4 (31)	

* Test non paramétrique de médiane comparant la distribution des âges selon le pronostic. ** Test exact de Fisher comparant les proportions de chaque variable selon le pronostic.

### Caractéristiques cliniques et paracliniques au diagnostic de la NL

Les comorbidités étaient peu fréquentes (n=5, 25%) et étaient représentées par l´hypertension artérielle (n=2), l´infection par le virus de l´immunodéficience humaine (n=1), l´infection par le virus de l´hépatite B (n=1) et l´albinisme (n=1). Le LES était diagnostiqué concomitamment à la NL chez 90% (n=18) des patients et 75% (n= 15) des patients consultaient dans les 3 mois qui suivaient l´apparition des symptômes rénaux (extrêmes 1-24 mois). Le syndrome néphrotique était le tableau clinique initial le plus fréquent dans les deux groupes (bon pronostic: 86%, n=6; mauvais pronostic: 46%, n=6; p=0,17). Les signes extra-rénaux étaient retrouvés chez tous les patients; les principaux signes étant les arthralgies (90%, n=18) et les lupus cutanés (65%, n=13). La médiane [IIQ] de la créatininémie de base était de 17 [10-22] mg/l et celle de la protéinurie de 5 [3, 4-7] g/g. Les anticorps anti-ADN natifs étaient retrouvés chez 10 (50%) patients et les anticorps anti-sm chez 3 (15%). Sept patients ont bénéficié d´une biopsie rénale et les classes III / IV actives étaient les plus fréquentes (72%, n= 5) (Tableau 2).

**Tableau 2:** caractéristiques cliniques et paracliniques au diagnostic des patients avec néphrite lupique colligés dans le service de néphrologie de l´Hôpital Général de Douala (Cameroun), de janvier 2015 à décembre 2020 (N=24)

Variables	Total (%) N=20	Bon pronostic (%) n=7	Mauvais pronostic (%) n=13	p*
**Signes rénaux**				
Hypertension artérielle de novo	10 (50)	3 (43)	8 (61,5)	0,999
Anurie	2 (10)	-	2 (15)	-
hématurie	11 (55)	5 (71,5)	9 (69)	0,999
Insuffisance rénale initiale	11(55)	3 (43)	9 (69)	0,611
Syndrome néphrotique	12 (60)	6 (86)	6 (46)	0,172
Syndrome néphritique aigue	5 (25)	1 (14)	4 (31)	0,578
Syndrome de gloméulonéphrite rapidement progressive	2 (10)	-	2 (15)	-
**Signes extra-rénaux**				
Fièvre au long cours	12 (60)	5 (71,5)	7 (54)	0,651
Lupus cutanés	13 (65)	6 (86)	7 (54)	0,318
Arthralgie	19 (95)	6 (86)	13 (100)	0,354
Neurolupus	10 (50)	3 (43)	7 (54)	0,999
Polyséite	10 (50)	5 (71,5)	5 (39)	0,631
Atteinte hématologique	11 (55)	5 (71,5)	6 (46)	0,371
Diagnostic concomitant du lupus	18 (90)	6 (86)	12 (92)	0,999
Phytothéapie	7(35)	2 (28,5)	5 (39)	0,633
**Biopsie rénale** (n=7)				
Classe 3 ou 4 active	5 (72)	3	2	-
Classe 4 chronique	1 (14)	-	1	-
Classe 5	1 (14)	-	1	-

* Test exact de Fisher comparant les proportions de chaque variable selon le pronostic.

### Caractéristiques thérapeutiques, devenir et survies

La corticothérapie a été initiée chez tous les patients à l´exception de la patiente qui présentait une classe IV chronique à l´histologie. Quatre patientes ont bénéficié uniquement de la corticothérapie: deux patientes ont refusé la mise sous immunosuppresseurs en raison des effets secondaires; une patiente était en rémission complète après les bolus de corticoïdes (la corticothérapie à dose régressive et l´hydroxychloroquine ont été maintenues avec persistance de la rémission complète) et une patiente qui était perdue de vue après le diagnostic et réadmise en IRCT après 16 mois. Au total, 75% (n=15) ont bénéficié d´un traitement d´induction et 30% des patients étaient en rémission après 6-12 mois (Tableau 3). Deux patients ont été perdus de vue entre 4 et 12 mois; ils étaient tous en rémission partielle.

**Tableau 3:** données thérapeutiques et devenir des patients avec néphrite lupique colligés dans le service de néphrologie de l´Hôpital Général de Douala (Cameroun), de janvier 2015 à décembre 2020 (N=24)

Variables	Total (%) N=20	Bon pronostic (%) n=7	Mauvais pronostic (%) n=13	p****
**Modalité de traitement**				
Corticothérapie monothérapie	4 (20)	-	4 (31)	**-**
Traitement induction (n=15)				
Corticoïde + cyclophosphamide*	12 (60)	5 (71,5)	7 (54)	0,656
Corticoïde + MMF	5 (25)	2 (28,5)	3 (23)	0,999
Traitement entretien (n=10)				
MMF	8	4 (57)	4 (31)	0,251
Azathioprine	2	0	2 (15,5)	**-**
**Autres**				
Hydroxychloroquinine	20 (100)	7 (100)	13 (100)	**-**
IEC/ARA	20 (100)	7(100)	13 (100)	**-**
**Evolution**				
Rémission complète	3 (15)	3(43)	-	**-**
Rémission partielle	3 (15)	3 (43)	-	**-**
Résistance	14 (70)	1 (14)	13 (100)	**<0,001**
≥ 2 poussées lupiques	10 (50)	-	10 (77)	**-**
Hémodialyse chronique **	5 (25)	-	5 (39)	-
IRCT	8 (40)	-	8 (61,5)	-
Décès***	11 (55)	-	11(85)	-

IEC = inhibiteur de l´enzyme de conversion, ARA II = antagoniste des récepteurs de l´Angiotensine II, MMF = Mycophénolate Mofétil, IRCT = insuffisance rénale chronique terminale. * Deux patients ont bénéficié d´une dose de Cyclophosphamide et ont continué le traitement d´induction avec le MMF. ** l´hémodialyse a été débutée au diagnostic chez 2 patients. *** Les décès incluent 3 sepsis, 3 neurolupus, 3 complications liées à IRCT, 1 mort subite et 1 tamponnade péricardique. **** Test exact de Fisher comparant les proportions de chaque variable selon le pronostic.

**Figure 1:**
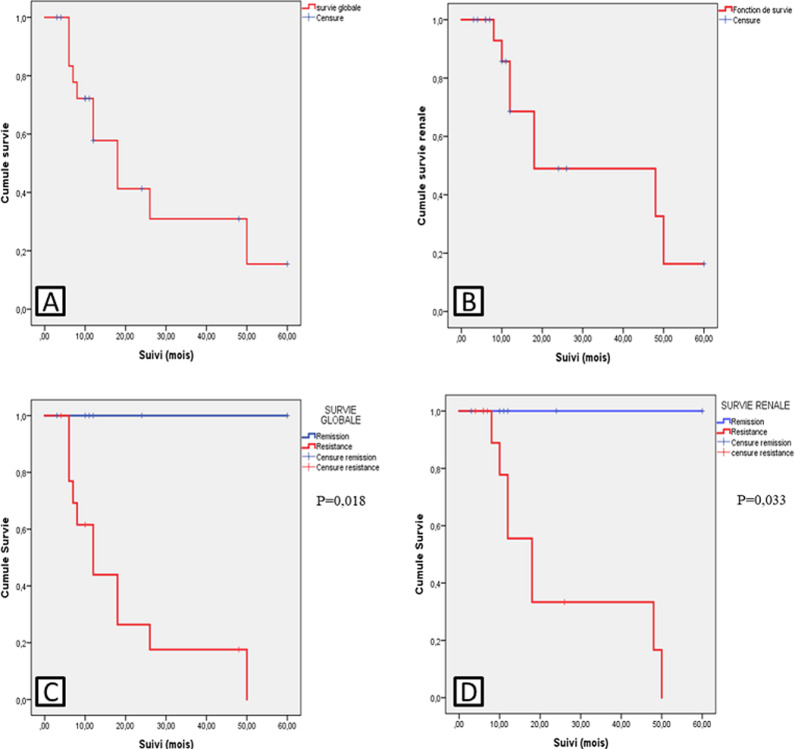
courbes de survie globale et rénale des patients avec néphrite lupique colligés dans le service de néphrologie de l’Hôpital Général de Douala (Cameroun), de janvier 2015 à décembre 2020 (N=24): A) survie globale B) survie rénale, C) survie globale selon la rémission (p=0,018) D) survie rénale selon la rémission (p=0,033)

L´hémodialyse a été initiée chez 5 des 8 patients ayant développé une IRCT au cours du suivi (2 refus de dialyse et une patiente vivant hors de la ville décédée d´un œdème aigue du poumon pendant son transfert vers l´HGD). La médiane [IIQ] de survie rénale était de 20 [50-11] mois avec des survies à 6, 12 et 24 mois respectivement de 93%, 68,6% et 49%. La mortalité globale était de 55% (n=11) et la médiane [IIQ] de survie globale de 18 [50-8] mois. Les survies globales à 6, 12 et 24 mois étaient respectivement de 83,3%, 57,8% et 31%. Les survies globale (p=0,018) et rénale (p=0,033) étaient plus faibles chez les patients qui n´étaient pas en rémission (Figure 1).

## Discussion

Vingt patients avec NL ont été identifiés sur une période de 5 ans. La NL était le principal mode de découverte du LES. Le syndrome néphrotique (60%) était le tableau clinique initial le plus fréquent. Les signes extra-rénaux étaient systématiques; les plus retrouvés étant les arthralgies et les lupus cutanés. L´insuffisance rénale initiale était notée chez 55% (n=11) des patients et les classes prolifératives actives étaient les plus fréquentes. Au total 11 (55%) patients sont décédés et 8 (40%) patients étaient en IRCT. A 12 et 24 mois, les survies rénale et globale étaient respectivement de 68,6% et 49%; 57,8% et 31%. La résistance au traitement était associée à des survies rénale et globale plus faibles. La NL est une complication fréquente du LES. Elle survient le plus souvent dans les 5 ans qui suivent la découverte du LES et constitue souvent le mode de révélation du LES [12]. Dans notre série, 90% des patients avec un LES étaient diagnostiqués au décours de l´atteinte rénale. En Afrique Subsaharienne, la prévalence de l´atteinte rénale au cours du LES varie entre 31 et 69% selon les définitions utilisées [7-10,13]. Le syndrome néphrotique, l´hypertension artérielle, l´insuffisance rénale et l´hématurie sont fréquemment décrits [8,9,14]; ce qui corroborent nos résultats.

La biopsie rénale est un élément clé de la prise en charge de la NL. En plus d´affirmer le diagnostic, elle permet d´évaluer le pronostic rénal [12]. Toutefois, elle reste difficile d´accès dans notre contexte (coût élevé, disponibilité du matériel, manque d´expertise locale) et seuls 7 patients ont pu bénéficier d´une biopsie rénale dans notre série. Bien qu´il n´existe pas de corrélation anatomoclinique au cours de la NL [15], l´hypertension artérielle, l´hématurie et l´insuffisance rénale sont plus couramment décrites dans la classe IV [15,16]. Ceci pourrait expliquer la fréquence élevée des formes proliférative et la sévérité du pronostic de la NL dans notre série. La NL était associée à un mauvais pronostic dans notre série: 55% des patients sont décédés, 40% ont évolués vers une IRCT avec des survies rénale et globale à 12 mois de 57,8% et 68,6% respectivement. Au cours de la NL, le risque d´IRCT à 5 ans est estimé à 11% dans les pays développés et la survie globale à 10 ans varie entre 92-98% [6,17]. Aux Etats-Unis, certains auteurs ont rapporté des survies rénales (68% vs 38%) et globale (81% vs 59%) à 10 ans plus faibles chez les patients noirs atteints de NL sévère comparées à celles des patients caucasiens [18]. En Afrique du Sud, la survie globale à 5 ans de la NL est estimée à 67% [19] et au Sénégal, le taux de mortalité est estimé entre 9-11% [8,9]. Différents facteurs peuvent expliquer les survies plus faibles observées dans notre série. Bien que la plupart des patients aient bénéficié d´un traitement d´induction, 70% d´entre eux présentaient une résistance au traitement après 6-12 mois.

De plus, les thérapies proposées dans le traitement des NL réfractaires telles que le Rituximab sont peu accessibles dans notre contexte et aucun patient n´a pu en bénéficier. L´immunosuppression était donc probablement insuffisante chez nos malades, expliquant une persistance de l´activité de la maladie et des poussées fréquentes (50%) qui sont reconnues comme des facteurs de progression vers l´IRCT au cours du LES [12]. L´atteinte rénale et l´ethnie noire sont également des facteurs de mauvais pronostic du LES décrits dans la littérature [3,4,12,18,20]. Des facteurs génétiques pourraient également être impliqués. En effet, les taux de mortalité rapportés au Sénégal varient entre 9-11% contre 55% dans notre série. Une étude récente au Ghana a relevé un pronostic plus sévère du LES chez les patients qui présentaient un génotype récessif des variants à haut risque de l´apolipoprotéine L1 (APOL1) avec un risque d´IRCT multiplié par 14 et un risque de décès à 12 mois multiplié par 13,6 [13]. Cette étude présente quelques limites. La faible taille de l´échantillon ne nous permet pas de généraliser nos résultats et ne nous a pas permis de faire des analyses de régression. Il s´agit d´une étude rétrospective et hospitalière. De ce fait, il est possible que seules les formes graves aient été répertoriées. Toutefois, il s´agit à notre connaissance de la première étude Camerounaise sur la NL. Elle fournit également des informations utiles sur le pronostic de la NL en Afrique Sub-saharienne où peu de données existent sur cette affection.

## Conclusion

La néphropathie lupique est un mode de révélation fréquent du lupus érythémateux systémique dans notre contexte. La survenue d´un syndrome néphrotique avec des signes extra-rénaux cutanés et articulaires doit faire discuter le diagnostic. Son pronostic reste sombre, la rémission est observée chez moins d´un tiers des patients malgré l´utilisation fréquente de Cyclophosphamide /Mycophénolate Mofétil et plus de moitié des patients décèdent ou sont en insuffisance rénale chronique terminale dans les deux ans qui suivent le diagnostic. Des études supplémentaires doivent être menées pour confirmer nos résultats, identifier des facteurs pronostiques et évaluer la contribution des facteurs génétiques.

### Etat des connaissances sur le sujet

- La néphrite lupique est une complication grave et fréquente du LES, elle constitue également un facteur de mauvais pronostic au cours du LES;
- Dans les pays développés, les survies globales et rénales à 5 ans sont au-delà de 90% mais elles seraient plus faibles chez les patients noirs;
- Peu de données sont disponibles sur la survie à court et long terme de la NL en Afrique Subsaharienne.

### Contribution de notre étude à la connaissance

- A notre connaissance il s´agit de la première étude sur la NL au Cameroun;
- Elle apporte des données sur le pronostic de la NL dans notre contexte et retrouve un pronostic beaucoup plus sévère que dans les pays développés: la rémission n´est notée que chez 1/3 des patients malgré l´utilisation du Cyclophosphamide et du Mycophénolate Mofétil et plus de la moitié des patients décèdent ou sont en IRCT dans les 24 mois.
